# Investigating the Pharmacological Mechanisms of Total Flavonoids from *Eucommia ulmoides* Oliver Leaves for Ischemic Stroke Protection

**DOI:** 10.3390/ijms25116271

**Published:** 2024-06-06

**Authors:** Jing Qin, Kewei Chen, Xiaomin Wang, Sirong He, Jiaqi Chen, Qianlin Zhu, Zhizhou He, Pengcheng Lv, Kun Chen

**Affiliations:** 1 The Joint Research Center of Guangzhou University and Keele University for Gene Interference and Application, School of Life Sciences, Guangzhou University, Guangzhou 510006, China; jingqin23@gmail.com (J.Q.); chenkewei55@163.com (K.C.); wangxiaomin34@163.com (X.W.); sironghe@163.com (S.H.); jiaqichen432@gmail.com (J.C.); zhuqianlin36@gmail.com (Q.Z.); kunchenju@gzhu.edu.cn (K.C.); 2 School of Chemistry and Chemical Engineering, Guangzhou University, Guangzhou 510006, China

**Keywords:** ischemic stroke, *Eucommia ulmoides* leaves, flavonoid, oxidative stress, network pharmacology

## Abstract

The aim of this study was to explore how the total flavonoids from *Eucommia ulmoides* leaves (EULs) regulate ischemia-induced nerve damage, as well as the protective effects mediated by oxidative stress. The cell survival rate was significantly improved compared to the ischemic group (*p* < 0.05) after treatment with the total flavonoids of EULs. The levels of reactive oxygen species (ROS), lactate dehydrogenase (LDH), and malondialdehyde (MDA) decreased, while catalase (CAT) and glutathione (GSH) increased, indicating that the total flavonoids of EULs can significantly alleviate neurological damage caused by ischemic stroke by inhibiting oxidative stress (*p* < 0.01). The mRNA expression level of *VEGF* increased (*p* < 0.01), which was consistent with the protein expression results. Meanwhile, the protein expression of *ERK* and *CCND1* increased (*p* < 0.01), suggesting that the total flavonoids of EULs could protect PC12 cells from ischemic injury via *VEGF*-related pathways. MCAO rat models indicated that the total flavonoids of EULs could reduce brain ischemia–reperfusion injury. In conclusion, this study demonstrates the potential mechanisms of the total flavonoids of EULs in treating ischemic stroke and their potential therapeutic effects in reducing ischemic injury, which provides useful information for ischemic stroke drug discovery.

## 1. Introduction

Cerebral infarction, namely ischemic stroke (IS), is a nervous system disease characterized by vascular obstruction [1,2]. About 15 million people suffer from strokes per year, of which the patients of IS account for 71% [3,4]. IS can also result in postoperative disability, Alzheimer’s disease, and even death [5,6,7]. However, antiplatelet drugs that protect IS from injury by preventing the formation of clots may cause symptoms of intracranial hemorrhage to appear [8,9,10]. More than 1000 potential neuroprotective agents have been discovered in experimental ischemic stroke, but only nearly 200 of them have been clinically tested [11,12]. The most useful therapy employs recombinant tissue plasmin activator (rt-PA). However, only 15% of patients can accept this treatment, since it can only be administered for a limited time after a stroke attack [13,14]. Additionally, rt-PA administration can lead to worsening inflammation, which is not conducive to stroke control and recovery [15]. Traditional herbal medicine (THM) has been demonstrated to be able to reduce early events of stroke, reduce the recurrence rate, and improve neurologic deficits [16,17]. Although various of mechanisms [18,19] have been proposed, it is still unclear why THMs have effects on IS treatment. Thus, clear clinical evidence and related comprehensive mechanisms need to be further explored for significant improvements in treatment efficacy.

Many medicinal herbs have been used to treat strokes for centuries in China [20,21]. *Eucommia ulmoides*, a unique plant species in China, is widely used in the processing of traditional Chinese medicine, especially its bark, but the comprehensive utilization rate of *E. ulmoides* shows insufficiency [22,23]. *E. ulmoides* contains 25 different flavonoids with different substituents, which have significant effects on treating cardiovascular diseases such as coronary heart disease and hypertension [24]. Studies have indicated that *E. ulmoides* leaves (EULs) contain more flavonoid components than bark and fruits [25]. Studying the active ingredients in EULs can improve the utilization rate of *E. ulmoides* resources and provide benefits for developing new drugs for treating IS.

Accordingly, this study aimed to analyze the relevant components and potential mechanisms of total flavonoids from *E. ulmoides* leaves (EULs) in the treatment of IS combined with network pharmacology. Then, experiments, including the extraction and component identification of total flavonoids from EULs, were conducted. Finally, their activity and mechanisms were explored in an in vitro ischemia model of PC12 cells.

## 2. Results

### 2.1. Network Pharmacology Prediction

#### 2.1.1. Compounds and Disease Target Prediction

After screening and deletion, six flavonoids were collected from relevant studies in the literature [23,26,27] and TCMSP (https://tcmsp-e.com/tcmsp.php, accessed on 6 April 2023), for which the common standards of oral bioavailability (OB) ≥ 30%, drug similarity (DL) ≥ 0.18, and Caco-2 ≥ 0 were used. These were kaempferol, quercetin, luteolin, baicalein, licochalcone A, and oroxylin A (Table 1).

**Figure 1:**
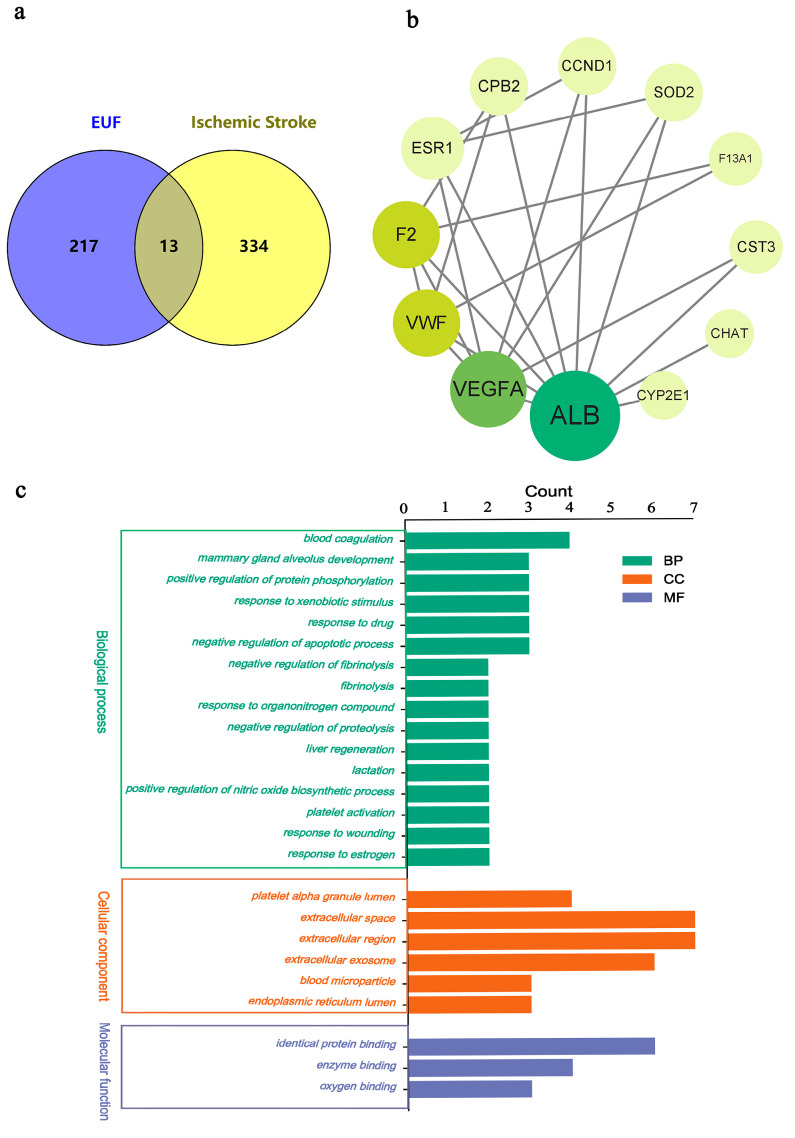

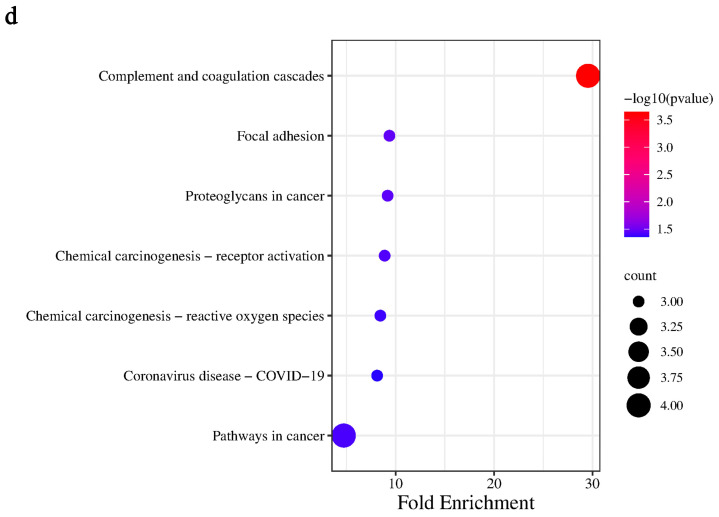
(**a**) Venny plot of the targets of IS and the total flavonoids of EULs. (**b**) Potential target interaction network of the total flavonoids of EULs in the treatment of IS. (**c**) Biological functional analysis of potential targets of the total flavonoids of EULs in the treatment of IS. (**d**) Bubble chart of KEGG enrichment key targets in the treatment of IS with the total flavonoids of EULs.

The Pharmmapper server was used to search for targets of the compounds above, and 217 potential compound targets were obtained after processing in the UniProt database. By searching the OMIM and GeneCards databases, a total of 334 IS targets were obtained. Finally, 13 intersection targets of drug compounds and disease were obtained through the Venny 2.1 software (Figure 1a).

Data from protein interaction networks were imported into Cytoscape (https://cytoscape.org/, accessed on 8 April 2023) to determine interaction networks between the target proteins. The nodes in the graph represent the target proteins, and the edges represent the correlations between the target proteins. Nodes with low correlation were removed, including 12 nodes and 23 edges. The main parameters used in the topological analysis were degree, proximity to centrality, and mesodegree centrality. In Figure 1b, the darker the color, the greater the degree value; the larger the circle, the greater the value of the mesocentricity; and the larger the font size, the greater the proximity to centrality.

#### 2.1.2. Pathway Enrichment Analysis

GO enrichment analysis was performed on 52 targets of the total flavonoids of EULs with the DAVID tool to further clarify the possible role of intersecting targets. The comparison entries for biological processes, molecular functions, and cellular components are shown in Figure 1c. The KEGG pathway enrichment analysis showed that the total flavonoids of EULs had a significant effect on seven biological pathways in IS. Their mechanisms of action mainly involve pathways in cancer, complement and coagulation cascades, focal adhesion, proteoglycans in cancer, chemical carcinogenesis–receptor activation, chemical carcinogenesis–reactive oxygen species, etc. (Figure 1d).

#### 2.1.3. Network Pharmacology Analysis

**Figure 2:**
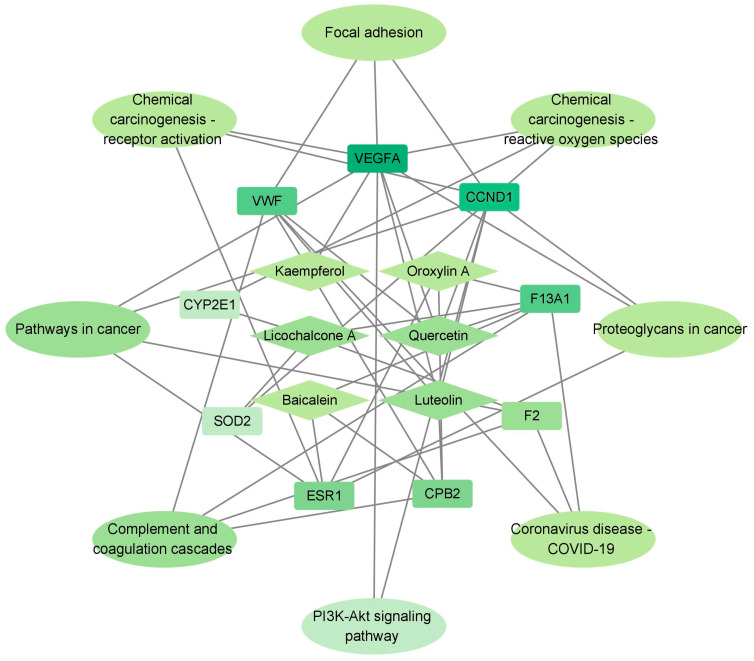
Compound-important target–pathway network.

In the protein interaction network, according to the size of the degree, the top nine targets combined with the subsequent KEGG pathway analysis were used to construct a compound–important target–pathway network (Figure 2). The network consisted of 23 nodes and 46 edges. And they were filtered according to their degree values: the darker the color, the higher the degree value, indicating the correlation was greater. Luteolin, quercetin, and licochalcone A were related to multiple targets, suggesting that they are important components in the treatment of IS.

#### 2.1.4. Molecular Docking

**Figure 3:**
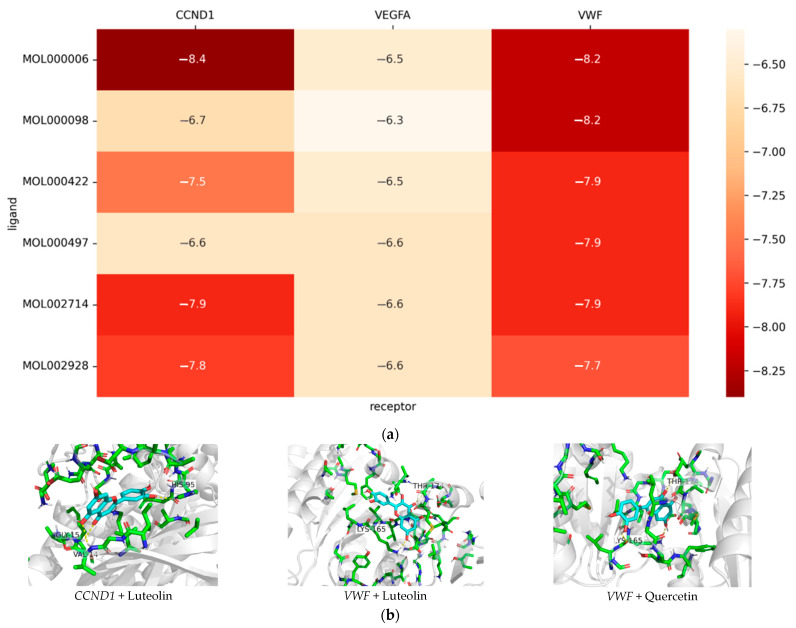
(**a**) Heat map of molecular docking results. (**b**) Simulation of protein–ligand docking.

Three important targets, namely *VEGF*A (PDB ID: 1MJV), *CCND1* (PDB: 2W9Z), and *VWF* (PDB: 7P4N), were selected and introduced into Autodock, and the six flavonoid compounds of EULs were screened for analysis (Figure 3a). If the binding energy was <−7 kJ/mol, the compound was considered to exhibit a strong binding activity to the target. There were 10 compounds with binding energies < −7 kJ/mol (55.6%). All compounds showed good binding activity to these three important targets. The docking results showed that the top three combinations with the highest docking scores for the three targets were luteolin (MOL000006 and *CCND1*), luteolin (MOL000006 and *VWF*), and quercetin (MOL000098 and *VWF*). These three pairs of target compounds were optimized using PyMOL 2.5.0, and the specific protein–ligand interactions in the docking are shown in Figure 3b.

### 2.2. Verification of Compounds

**Figure 4:**
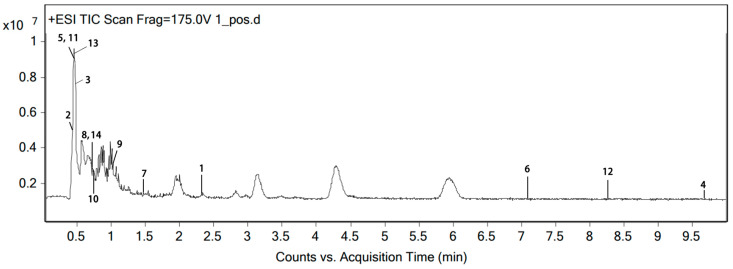
Total ion chromatogram (TIC) of the total flavonoids extracted from EULs in positive-ion mode.

The flavonoid components in the total flavonoids extracted from the EULs were identified using Agilent Masshunter B.08.00 software. A molecular weight calculator in this software was used to calculate the *m*/*z* of the target compounds in different modes. Then, the chromatogram from the TIC graph was extracted, EIC was selected as the type, the *m*/*z* values were input separately to carry out the calculation. Possible compounds were returned to the mass calculator in the molecular weight calculator to compare their mass, and, finally, 14 flavonoids were identified from the total flavonoids of the EULs (Figure 4), which included the 6 flavonoids screened using network pharmacology above. These are displayed in Table 2. The total relative content of the six flavonoids screened through network pharmacology was close to half of all of the flavonoids.

### 2.3. Activity Assay

#### 2.3.1. Effects of Total Flavonoids of EULs on Cell Viability after Ischemia-Induced PC12 Cell Injury

**Figure 5:**
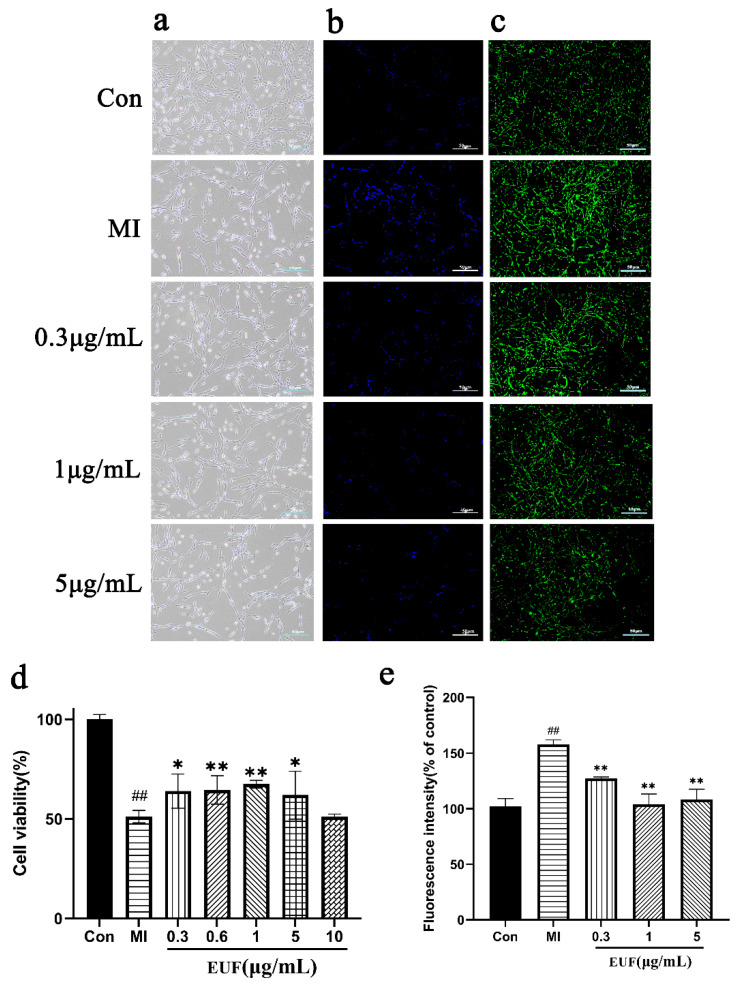
(**a**) Effects of the total flavonoids of EULs on PC12 ischemia-induced injury. (**b**) Fluorescence micrograph of cells after Hoechst 33258 nuclear staining followed by fluorescence imaging to detect apoptosis (×100). (**c**) Changes in ROS fluorescence intensity of PC12 after ischemia-induced injury with different concentrations of total flavonoids from EULs (100×). (**d**) Effects of treatment with total flavonoids of EULs at different concentrations for 24 h on cell viability after ischemia-induced PC12 cell injury. (**e**) Analysis of ROS fluorescence intensity of PC12 ischemia-induced injury with different concentrations of total flavonoids from EULs. All scale bars are 50 μm. Data represent the mean ± standard deviation, and the graphs show representative data from three independent experiments. Compared with the control group, ## *p* < 0.01; compared with the myocardial infarction group (MI), ** *p* < 0.01, * *p* < 0.05.

The cell viability assays were performed using CCK-8 (cell count kit-8) assay kits and the results showed that the cell viability of the ischemic group was significantly lower than that of the blank control group (51.10% ± 3.11%). Five concentration groups (0.3, 0.6, 1, 5, 10 μg/mL) were set, and the cell viability showed a trend of concentration inhibition dependent on the drug dose: as the drug concentration increased, the cell viability first increased and then decreased (Figure 5a,d). The maximum cell viability was reached when the drug concentration was 1 μg/mL (67.41% ± 1.94%), and the difference was statistically significant (*p* < 0.05). The activity was significantly reduced when the concentration was 10 μg/mL, indicating that 10 μg/mL was toxic to the cells.

#### 2.3.2. Total Flavonoids of EULs Reduce Ischemia-Induced Apoptosis in PC12 Cells

To further verify the protective effect of the total flavonoids of EULs on PC12 cells, the effect of treatment with the total flavonoids of EULs on the apoptosis of PC12 cells was evaluated. The morphological characteristics of the nuclear chromatin of apoptotic cells were detected using the Hoechst 33258 staining method (Figure 5b). Compared with the blank control group, apoptosis was increased in the ischemic group, with nuclear coalescence, rupture, cell membrane bubbles, the appearance of apoptotic vesicles, and a large amount of blue fluorescence.

#### 2.3.3. Effects of Total Flavonoids of EULs on ROS after Ischemia-Induced PC12 Cell Injury

The ROS results showed that the green fluorescence intensity of the ischemic group was significantly higher than that of the blank control group, and the number of reactive oxygen species decreased after drug administration treatment. After analyzing the fluorescence intensity (Figure 5c), the results showed that the best inhibition of reactive oxygen species accumulation in cells was achieved at a drug concentration of 1 μg/mL (Figure 5e).

#### 2.3.4. Effects of Total Flavonoids of EULs on LDH, CAT, MDA, and GSH after Ischemia-Induced PC12 Cell Injury

**Figure 6:**
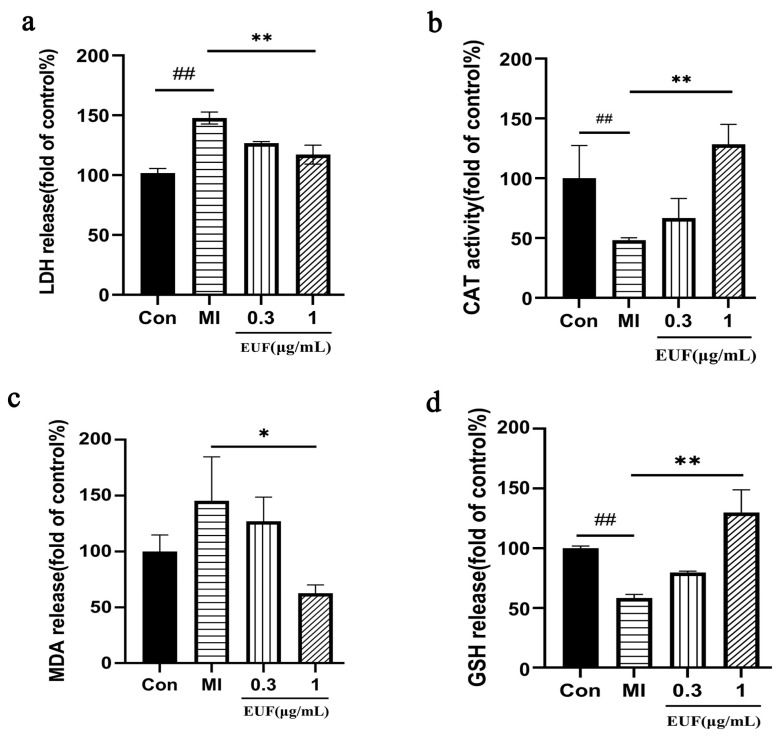
Effects of the total flavonoids of EULs on LDH, CAT, MDA, and GSH after ischemia-induced PC12 cell injury. ## *p* < 0.01, ** *p* < 0.01, * *p* < 0.05.

The results (Figure 6a) showed that the release of LDH in the treatment group was significantly lower than that in the ischemic group, especially when the concentration of the total flavonoids of EULs was 1 μg/mL. The activity of CAT (Figure 6b) was significantly decreased after ischemia (*p* < 0.01) and increased after treatment with the total flavonoids of EULs. The analysis showed that the release of MDA (Figure 6c) and the release of GSH (Figure 6d) were significantly decreased after treatment with the total flavonoids of EULs compared with that of MI (*p* < 0.01). The above results indicate that the EUL flavonoid treatment had significant effects on protecting PC12 cells with ischemia-induced injury from oxidative stress.

#### 2.3.5. Effects of Total Flavonoids of EULs on *VEGF* Gene Expression after Ischemia-Induced PC12 Cell Injury

**Figure 7:**
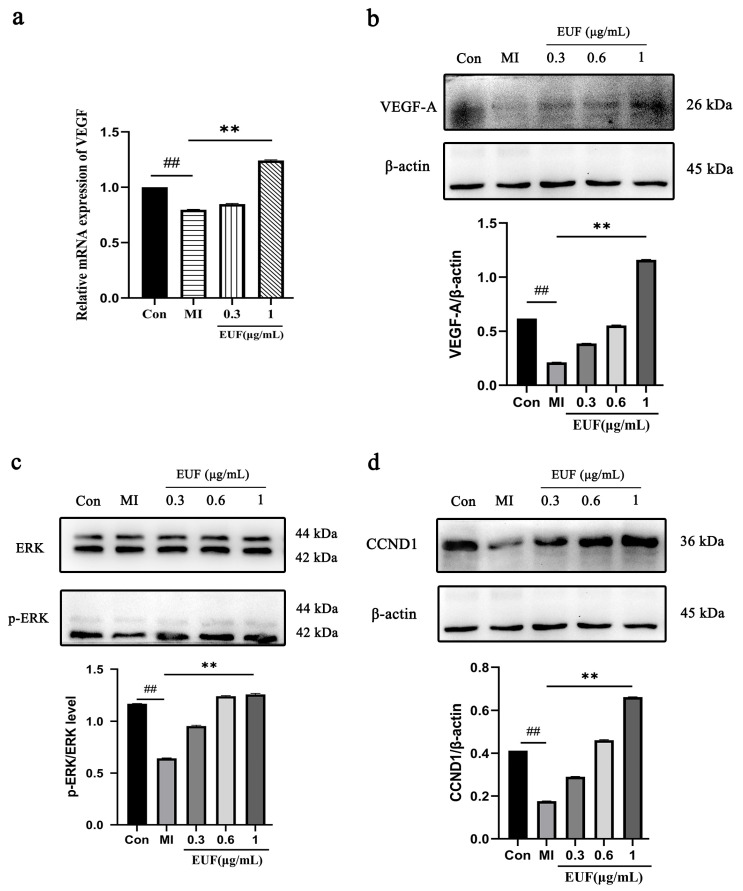
(**a**) mRNA expression of *VEGF* after ischemia-induced PC12 cell injury. (**b**) Effects of treatment with total flavonoids of EULs on the levels of *VEGF-A* after ischemia-induced PC12 cell injury. (**c**) Effects of treatment with total flavonoids of EULs on the levels of *p-ERK* after ischemia-induced PC12 cell injury. (**d**) Effects of treatment with total flavonoids of EULs on the levels of *CCND1* after ischemia-induced PC12 cell injury. ## *p* < 0.01, ** *p* < 0.01.

Brain tissue and neural repair after IS can be promoted by *VEGF*. As shown in Figure 7a, the mRNA expression of the *VEGF* in the ischemic group was significantly lower than that in the control group (*p* < 0.01), which was significantly increased after 24 h treatment with the total flavonoids of EULs (*p* < 0.01). The level of *VEGF-A* after the EUL flavonoid treatment in the ischemic PC12 cells also proved this (Figure 7b). According to the research above, *VEGF* played an important role in repairing the IS damage.

#### 2.3.6. Effects of Total Flavonoids of EULs on the Levels of *p-ERK* and *CCND1* after Ischemia-Induced PC12 Cell Injury

To further investigate the signaling mechanisms underlying how the total flavonoids of EULs affect ischemia-induced injury and confirm the results of the network pharmacology analysis, the levels of *p-ERK* and *CCND1* after EUL flavonoid treatment in the ischemic PC12 cells were analyzed (Figure 7c,d). The levels in the ischemic group were significantly decreased compared with control group, which were increased after treating the PC12 cells with the total flavonoids of EULs (*p* < 0.01). The results above suggest that the total flavonoids of EULs probably protected the ischemic PC12 cells from injury though the focal adhesion pathway.

#### 2.3.7. Effects of Total Flavonoids of EULs on Brain Injury Induced by Ischemia–Reperfusion in Rats

**Figure 8:**
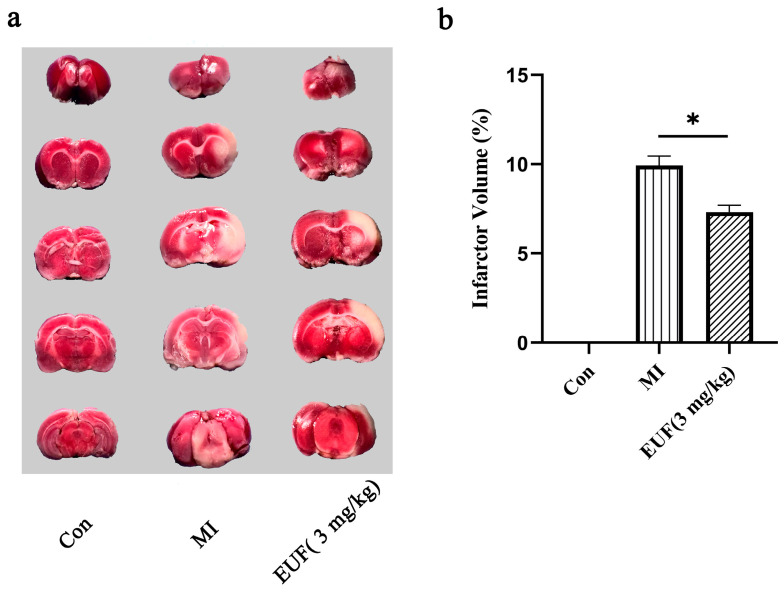
(**a**) Brain section stained with 2,3,5-triphenyltetrazolium chloride (TTC) in different groups. The white area represents the infarcted part, and the red area represents the normal part. (**b**) Quantitative analysis of cerebral infarct volume, * *p* < 0.05.

The volume of cerebral infarction after ischemia–reperfusion injury was measured using 2,3,5-triphenyltetrazolium chloride (TTC) staining (Figure 8a). Almost no infarcted areas were observed in the brain tissue samples of the blank control group. However, a larger infarcted area was observed in the model group. Compared to the model group, the infarcted area was reduced in the group treated with the total flavonoids of EULs. A statistical data analysis of the cerebral infarction volume provided specific evidence that administration of the total flavonoids of EULs reduced the infarction volume (Figure 8b).

#### 2.3.8. Pathological Observation of the Effects of Total Flavonoids of EULs on Hippocampal Injury Induced by Ischemia–Reperfusion in Rats

**Figure 9:**
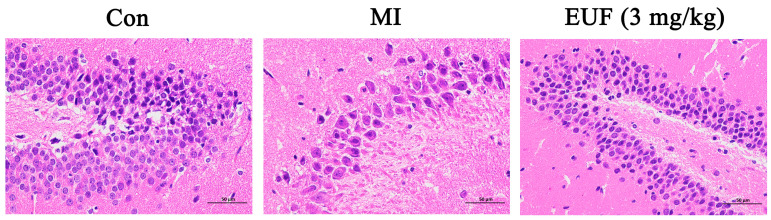
Protective effects of total flavonoids of EULs (3 mg/kg) on hippocampal injury induced by ischemia–reperfusion in rats.

The HE staining method was used to observe the damage of hippocampal tissue and nerve cells. Observing the HE staining results for hippocampal tissue slices of the rat ischemic model in Figure 9, it can be seen that compared with the sham surgery group, the ischemic group had widened gaps around the cells, which contained many vacuoles of varying sizes, loosely arranged cells, uneven cytoplasmic staining degrees, deformed nuclear folds, obvious apoptotic characteristics, and a reduced number of neurons. Compared with the ischemic group, the EUL flavonoid group showed a shortened pericellular gap, gradually clearer cell contours, and an increase in the number of neurons, indicating that the total flavonoids of EULs can protect neurons from ischemic damage.

## 3. Discussion

In this paper, network pharmacology was used to predict the function of the total flavonoids of EULs on ischemia-induced PC12 cells. Compared with the extraction and analysis of compounds before network pharmacology, the method in this study provided a better solution for conducting research on natural products. Studying traditional plants can not only find more methods to conquer toxicity and drug resistance but can also make full use of natural resources. To the best of our knowledge, this is the first work to study the effects of the total flavonoids of EULs on IS.

As one of the most common components of plants, flavonoids play an important role both in plant metabolism and in human health. According to our analysis of network pharmacology, the total flavonoids of EULs did work in treating IS. Luteolin, quercetin and licochalcone A in the EULs were found to be the most relevant flavonoid compounds for ischemia therapy. *VEGF-A*, *CCND1*, and *VWF* were the key targets in curing ischemic injury, which we demonstrated through the molecular docking analysis. UPLC-Q-TOF-MS was used to check out whether the total flavonoids of the EULs had the flavonoids that we needed. This made our work more logical and meaningful. Though about 19% of the total flavonoids of the EULs were other ingredients, these took little part in our analysis and so could be neglected. Furthermore, our experiments were designed based on the concentration of certain flavonoids in the total flavonoids of the EULs, enabling regularity in the biological experiments. The ischemic stroke model showed different cell activities with different concentrations. As the absolute flavonoid content increased, the biological activity changed at the same time. Thus, we considered that the studied flavonoids in the total flavonoids of the EULs were mostly responsible for the protection effects observed.

PC12 cells have been used previously to mimic some of the properties of in vivo brain ischemia–reperfusion injury and have been instrumental in identifying common mechanisms such as calcium overload, redox potential, lipid peroxidation, and MAPK modulation [28]. According to our further study, we found that the total flavonoids of EULs can significantly improve the symptoms of ischemia-induced injury in PC12 cells. Flavonoids have proven to be a sort of antioxidant component, which can be widely found in EULs. Flavonoid antioxidant activity can prevent damage caused by free radicals through scavenging reactive oxygen species (ROS), activating antioxidant enzymes, inhibiting oxidases, and reducing α-tocopheryl radicals [29]. After treatment with the total flavonoids of EULs, the release of ROS was significantly decreased, indicating that the total flavonoids of EULs may work as antioxidants. Our study showed that the antioxidant enzyme CAT plays an important role in the defense against oxidative stress. The levels of cell damage and oxidative stress indicators, including MDA and LDH, were significantly decreased after treatment with the total flavonoids of EULs compared with the ischemic group. As the most important endogenous small-molecule antioxidant, GSH’s activity was also improved, and the cells were protected from ischemia-induced cytotoxicity.

In order to test the results of the network pharmacology analysis and predict mechanisms, subsequent experiments using Real-Time Fluorescence Quantitative PCR (RT-PCR) and Western blot analysis were designed. The RT-PCR results showed that treatment with the total flavonoids of EULs improved the gene expression of *VEGF* compared with the ischemic group, proving the reliability of network pharmacology analysis. *VEGF* repairs brain damage and has a neurotrophic effect in the central nervous system and can promote the proliferation and differentiation of neural stem cells, neural precursor cells, and even glial cells. *MAPK/ERK* is the downstream target of *VEGF* [30]. The upregulation of *VEGF* expression can promote the proliferation and migration of vascular endothelial cells, induce neovascularization, and restore cerebral microvascular circulation [31]. This study related the results of signal pathway prediction and molecular docking to successively verify the protein expressions of *p-ERK* and *CCND1* (Cyclin D1), proving that the total flavonoids of EULs can recover cell ischemic injury by regulating the protein expressions of *ERK* and *CCND1*. In animal experiments, the oral administration of flavonoids is more relevant to the physiological state, which is also the experimental basis for developing them into food-homologous drugs. Although the total flavonoids of EULs may have different pathways of action in in vitro and in vivo experiments, they both played a role in alleviating ischemic injury. The total flavonoids of EULs have a certain value for practical applications in protecting nerve cells from ischemic damage. The results of TTC staining and HE staining from our ischemia–reperfusion model in rats indicated that the total flavonoids of the EULs significantly reduced ischemia-induced injury, demonstrating their potential application in ischemic stroke therapy.

## 4. Materials and Methods

### 4.1. Animals

Healthy adult SPF clean-grade SD rats, 250–310 g, were purchased from Jianggao Wenyi Experimental Farm, Guangzhou, China. All experimental operations strictly complied with the “Guiding Opinions on Treating Experimental Animals Well” (China Ministry of Science and Technology 2006) and the ethical regulations on experimental animals outlined by Guangzhou University.

### 4.2. Reagents and Materials

Rutin was purchased from Qiyun Biotechnology Co., Ltd. (Guangzhou, China). AB-8 microporous resins were purchased from Huideyi Technology Co., Ltd. (Beijing, China). Sephadex^®^ LH-20 was purchased from GE Healthcare Bio-Sciences AB (Uppsala, Sweden). EtOH was obtained from Merck (Darmstadt, Germany). AlCl_3_, Na(NO_3_)_2_, Al(NO_3_)_3_, and NaOH were purchased from the Damao Chemical Reagent Factory (Tianjin, China). PC12 cells were obtained from the First Affiliated Hospital of Sun Yat sen University as a gift. The CCK-8, BCA, LDH, and ROS reagents were purchased from Beyotime Biotechnology Co., Ltd. (Shanghai, China). The PrimeScript^TM^ RT reagent kit was purchased from TaKaRa Biotechnology Co., Ltd. (Osaka, Japan). *VEGF-A*, Cyclin D1 antibody, p-p44/42 MAPK (*p-Erk*1/2) rabbit mAb, anti-rabbit IgG, and HRP-linked antibody were purchased from Youningwei Biotechnology Co., Ltd. (Shanghai, China). All other reagents were of analytical grade.

The EULs were harvested in December 2021 from Henan, China, and authenticated by Prof. Chen, Department of Biopharmaceuticals, College of Life Sciences, Guangzhou University, the voucher specimen of which (ID 20211208 for *E. ulmoides* leaves) was deposited at the authors’ laboratory.

### 4.3. Network Pharmacology

#### 4.3.1. Active Compounds and Compound Targets

Active compounds were obtained according to relevant studies in the literature and TCMSP (https://tcmsp-e.com/tcmsp.php, accessed on 6 April 2023), for which the commonly used standards of oral bioavailability (OB) ≥ 30%, drug similarity (DL) ≥ 0.18, and Caco-2 ≥ 0 were used.

The mol2 format files of the active compounds obtained above were uploaded to the Pharmaper server (http://www.lilab-ecust.cn/pharmmapper/, accessed on 6 April 2023) to obtain the predicted targets. The searched targets were imported into the UniProt database (https://www.uniprot.org, accessed on 7 April 2023). Relevant targets for IS were collected through OMIM (https://www.omim.org/, accessed on 6 April 2023) and GeneCards (https://www.genecards.org, accessed on 6 April 2023). Venny 2.1 software was used to match the active compound targets of the total flavonoids of EULs with the relevant targets of IS, selecting overlapping targets as the targets of the total flavonoids of EULs for the treatment of IS.

#### 4.3.2. Network Construction and Mechanism Analysis

String (https://string-db.org/, accessed on 8 April 2023) was used to process the relevant targets and obtain the final protein interaction network. The data of the compound protein interaction network were inputted into Cytoscape and used to construct different networks: a composite target network and a compound-important target–pathway network.

GO biological process and KEGG metabolic pathway enrichment analyses were conducted through DAVID database (http://david.abcc.ncifcrf.gov/, accessed on 8 April 2023) retrieval and conversion. *p* < 0.05 was used to identify significant biological processes. The online mapping website bioinformatics (http://www.bioinformatics.com.cn/, accessed on 8 April 2023) was used to visualize the results.

#### 4.3.3. Molecular Docking Analysis

Potential active ingredients could be screened out and the binding activity of the total flavonoids of EULs with important targets could be verified through molecular docking. The small molecular structures of the compounds collected earlier were obtained from the TCMSP database and saved as mol2 files. All of the protein ligands with important target-protein crystal structures were obtained from the Uniprot protein database (https://www.uniprot.org, accessed on 10 April 2023). Finally, the two types of data were imported into the Automatic Dock 4.4.6 for molecular docking, and the docking score was used for the evaluation criteria.

### 4.4. The Process of Obtaining the Total Flavonoids of EUL

#### 4.4.1. Determination of Flavonoid Content

The content of total flavonoids in the EULs was detected at 510 nm using ultraviolet and visible (UV-Vis) spectrophotometry [32]. A rutin standard curve was established. The relationship between absorbance A and concentration C was A = 18.718C-0.0077 (R^2^ = 0.9991), which showed a good linear relationship between 0 and 0.05 mg/mL. Subsequently, the flavonoid content in each sample could be calculated using this standard curve.

#### 4.4.2. Extraction and Isolation

The dry EUL powder (5.0 g) was extracted with 60% ethanol and refluxed twice at 80 °C for 90 min each time. The extract was collected together and concentrated using a rotary evaporator. The concentrate was dissolved and suspended in 10 mL water. Subsequently, it was purified with AB-8 macroporous resin column chromatography, eluted with different concentrations of ethanol (0% and 90%). The 90% ethanol-eluted fraction was concentrated and dissolved with 70% ethanol to 20 mg/mL. Finally, the concentrate was subjected to a Sephadex LH-20 column eluted with 70% ethanol to yield the total flavonoids from the EULs. About 0.01 g of the total flavonoids of the EULs was obtained for following research after being dried, of which the total flavonoids content was 81%. All the steps above were performed through experiments shown in the patent (CN 116098930 B).

### 4.5. UPLC-Q-TOF-MS Analysis

The Agilent 1290 Series UPLC System (Agilent, Santa Clara, CA, USA) was used for chromatographic evaluation. Samples were separated on C18 columns (2.1 × 50 mm, 1.8 μm, Agilent, Santa Clara, CA, USA) with methanol solvents in the mobile phase. Flow rate: 0.3 mL/min; column temperature: 30 °C; injection volume: 4 μL. Then, the separated components were passed through the Agilent 645 Q/TOF mass spectrometer equipped with the ESI interface (Agilent, Santa Clara, CA, USA), for which the operating parameters were as follows: dry N_2_ gas flow rate: 10 L/min; temperature: 375 °C; nebulizer: 30 psig; capillary: 0.066 μA; skimmer: 45 V; OCT RfV: 750 V; fragments: 175 V; MS data acquisition mode: MS (seg); max precursor per cycle: 3; acquisition time: 333.3 ms/spectrum. Samples were analyzed in positive-ion mode and mass spectrometry data were recorded in the range of 50–1700 *m*/*z*. Internal mass calibration was performed using reference masses of 121.0509 (purine) and 922.0098 (HP-0921) during operation in positive-ion mode.

### 4.6. Ischemic Protection Effect Assay

#### 4.6.1. Cell Culture and Transfection

PC12 cells were cultured in high-glucose DMEM containing 10% FBS+2% double antibodies (100 U/mL penicillin and 100 mg/mL streptomycin), and placed in a 37 °C, 5% CO_2_ incubator. The fluid was changed every 1–2 days and passed every 2–3 days. Cells in the logarithmic growth phase were used for experiments.

#### 4.6.2. Establishment and Grouping of Ischemic Models

Cells in the logarithmic growth phase were inoculated at 1 × 10^5^ cells/well into different culture plates with high-glucose DMEM containing 10% FBS+2% dual antibody for 24 h, and the old culture solution was discarded. The cells were washed once with PBS and divided into a blank control group, an ischemic group, and a post-ischemia-treatment group (0.3, 1, and 3 µg/mL of the total flavonoids of EULs). The concentration of the total flavonoids of EULs was the absolute content of the total flavonoids. For example, 1 μg/mL total flavonoids of EULs indicated that a 1 ml solvent system contained 1 μg of total flavonoids. Except for the blank group, where normal culture medium was added, all other groups were treated with the same volume of ischemic solution (buffer containing 20 mM 2-DOG, 20 mM sodium lactate, and 2.5 mM Na_2_S_2_O_4_). After 20 min of treatment, the ischemic model was established.

#### 4.6.3. Cell Viability Assay

For the cell viability assay, 1 × 10^5^ cells were inoculated into 96-well plates at a volume of 100 µL per well. Each group consisted of 6 parallel wells. After 24 h of dosing treatment, 10 µL of CCK-8 solution was added to each well and they continued to be incubated in the incubator for 1 h. They were measured with an enzyme-linked immunosorbent assay at 450 nm absorbance.

#### 4.6.4. Hoechst 33258 Nuclear Staining

For the Hoechst 33258 nuclear staining, 1 × 10^5^ cells were inoculated into a 6-well plate at a volume of 2 mL per well. The cells were treated according to Section 4.6.2. After 24 h of drug culture, they were washed once with PBS and fixed with 4% paraformaldehyde. The paraformaldehyde was discarded after 20 min, and 0.5 µg/mL of the Hoechst 33258 staining solution was added in darkness. After 25 min of staining, the cells were washed three times with PBS, and they were observed and photographed with a 100× magnification microscope.

#### 4.6.5. ROS, LDH, CAT, MDA, and GSH Analyses

For these analyses, 1 × 10^5^ cells were inoculated into a 6-well plate at a volume of 2 mL per well. The cells were treated according to Section 4.6.2. After 24 h of drug culture, they were washed once with PBS. DCFH-DA was diluted with culture medium in a ratio of 1000:1 and 1 mL of diluted DCFH-DA was added into each well. After incubation for 20 min in the cell incubator, the cells were washed three times with serum-free cell culture medium, and then observed and photographed under a laser confocal microscope for analyzing the fluorescence intensity of the ROS.

Then, 1 × 10^5^ cells were inoculated into 96-well plates with 200 µL per well, and 6 duplicate wells were created in parallel for each group. After incubation for 24 h, 120 µL of the supernatant was transferred to a new 96-well plate and an LDH test kit was used to detect the LDH activity. CAT, MDA, and GSH activities were all tested according to the corresponding kits.

#### 4.6.6. Real-Time Fluorescence Quantitative PCR Assay

The cells were inoculated into 6-well plates. After 24 h of drug cultivation, the culture medium was discarded, and the cells were washed once with PBS at 4 °C. Total RNA was extracted and 1uL of each sample was used to determine the concentration.

A PrimeScript^TM^ RT reagent kit with gDNA Eraser was used to reverse the transcription to obtain uniform cDNA. The PCR reaction solution was prepared on ice and a real-time PCR reaction was performed. Gene expression in the PC12 cells after treatment was measured by pre-denaturing the cells at 95 °C for 30 s, followed by a PCR reaction at 95 °C for 5 s and finally at 4 °C for 30–60 s. The *VEGF* primers were 5′-AGCGAGAACAGCCCAGAAG-3′ and 5′-GACGGACAGACAGACAGACA-3′.

#### 4.6.7. Western Blot Analysis

The cells were inoculated into 6-well plates. After 24 h of drug cultivation, the culture medium was discarded, and the cells were washed 3 times with PBS at 4 °C. After being lysed, collected, rested on ice for 30 min, and centrifuged at 13,200 rpm, 4 °C, for 15 min, the supernatant was collected and determined using a BCA kit to obtain the loading volume. The protein sample with the loading buffer was denatured in boiling water at 99 °C for 5 min.

Then, 10 µg of protein was loaded and subjected to SDS-PAGE electrophoresis, and transferred to a PVDF membrane. After being blocked with 5% milk for 1 h, a primary antibody (1:1000) was incubated overnight at 4 °C, and a secondary antibody of sheep anti-rabbit IgG (1:5000) was applied at room temperature for 1.5 h. The ECL chemiluminescence reagent was added and the bands were analyzed and photographed using a Bio Light imaging system (Bio-Rad, Hercules, CA, USA).

#### 4.6.8. Establishment of MACO Model in Rats

The adult SD rats were randomly divided into three groups: the sham surgery group, the model group, and the group treated with the total flavonoids of EULs (3 mg/kg), with 4 rats in each group. The drug was given every day for 3 days to the model group, while the others received normal saline. Then, 30 min after the last administration, MCAO modeling was performed for all groups except the sham surgery group. The rats were anesthetized with 10% chloral hydrate at a depth of 4 mL/kg. After disinfection, they were incised in the middle of the neck and bluntly separated to expose the right common carotid artery (CCA), internal carotid artery (ICA), and external carotid artery (ECA). The proximal and distal ends of the CCA were separated and ligated, and the distal end of the ECA was also ligated. A 0.26 mm thread plug was inserted at the proximal end of the ECA ligation site, and threaded through the ICA until the middle cerebral artery (MCA) was blocked. The skin was sutured after fixation with thread, and the thread plug was removed for 2 h before reperfusion. The sham surgery group did not have thread plugs inserted but the other steps were the same. After 24 h, the rats were euthanized through neck dislocation and their brains were immediately removed through craniotomies.

#### 4.6.9. TTC Staining

The brains of the rats were frozen at −80 °C for 5 min and then cut into 5 sections, each 2 mm thick. Each slice was immediately stained with a 1% 2,3,5-triphenyltetrazolium chloride (TTC) solution at 37 °C for 30 min. The infarcted area was not stained, while the normal area was stained red. Then, each slice was photographed and the infarcted area of the brain was calculated using ImageJ 1.54.

#### 4.6.10. HE Staining

The frozen hippocampi of the rats were cut into 4–8 µm sections, dewaxed in xylene for 5–10 min, and dehydrated with 100%, 95%, 85%, and 70% alcohol for an average of 2–5 min. Then, they were transferred to a hematoxylin staining solution with distilled water and stained for 5–15 min. Excess dye solution on the slide was washed, using 0.5 to 1% hydrochloric acid alcohol for differentiating. Differentiation was terminated when the cell nucleus and chromatin in the cell nucleus were observed under the microscope clearly. Then, each slide was rinsed with tap water for 15–30 min, and a 1% ammonia water solution was added to return it to blue. Next, it was rinsed with distilled water, stained with 0.1–0.5% eosin dye for 1–5 min, and dehydrated with 70%, 85%, 95%, and 100% alcohol for an average of 2–3 min in all steps. Excess xylene around the slices was wiped off and an appropriate amount of neutral gum was used to seal them.

#### 4.6.11. Statistical Analyses

All data were plotted as the mean ± standard deviation. The statistical analyses were performed with GraphPad Prism9 software. A one-way analysis of variance was applied for assessing different levels of significance between the multiple groups of samples. The criterion for all significance tests was *p* < 0.05.

## 5. Conclusions

In this study, network pharmacology was used to analyze the key components of flavonoids in EULs, concluding that *VEGF*, *CCND1*, and *ERK* were the most important targets to verify. Then, a UPLC-Q-TOF-MS rapid analysis system confirmed the existence of the flavonoids that we were interested in. Third, it was shown that the protective effects of the total flavonoids of EULs were mediated by a mechanism dependent on antioxidants and the activation of *VEGF*. In summary, the total flavonoids of EULs are a promising natural product that could be used to prevent IS.

## Figures and Tables

**Table 1:** Screening of effective components of total flavonoids in EULs.

MOL-ID	Ingredient	Chemical Structure	Molecular Formula	OB (≥30%)	DL (≥0.18)
MOL000422	Kaempferol	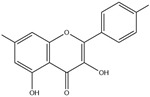	C_15_H_10_O_6_	41.88	0.24
MOL000098	Quercetin	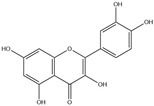	C_15_H_10_O_7_	46.43	0.28
MOL000006	Luteolin	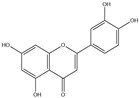	C_15_H_10_O_6_	36.16	0.25
MOL002714	Baicalein	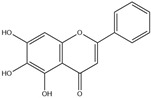	C_15_H_10_O_5_	33.52	0.21
MOL000497	Licochalcone A	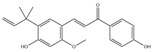	C_21_H_22_O_4_	40.79	0.29
MOL002928	Oroxylin A	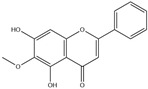	C_16_H_12_O_5_	41.37	0.29

**Table 2:** Characterization of the total flavonoids extracted from EULs via UPLC-Q-TOF-MS analysis in positive-ion mode.

No.	RT (min)	*m*/*z*	Formula	Ingredient	Mode	Error (ppm)	Ref.
1	2.34	325.0109	C_15_H_10_O_6_	Kaempferol	[M + K]^+^	−0.01	[26]
2	0.423	303.0499	C_15_H_10_O_7_	Quercetin	[M + H]^+^	0.1	[26]
3	0.489	327.0863	C_15_H_10_O_6_	Luteolin	[M + C_3_H_5_]^+^	0.04	[23]
4	9.658	293.0420	C_15_H_10_O_5_	Baicalein	[M + Na]^+^	0.15	[23]
5	0.445	356.1856	C_21_H_22_O_4_	Licochalcone A	[M + NH_4_]^+^	−0.1	[23]
6	7.091	307.0577	C_16_H_12_O_5_	Oroxylin A	[M + Na]^+^	−0.02	[23]
7	1.49	815.2604	C_32_H_38_O_21_	Quercetin glycoside 1	[M + C_4_H_9_]^+^	0.04	[23]
8	0.728	667.1869	C_27_H_30_O_17_	Quercetin glycoside 2	[M + C_3_H_5_]^+^	−0.04	[23]
9	1.023	653.2076	C_26_H_28_O_16_	Quercetin 3-O-sambubioside	[M + C_4_H_9_]^+^	0.02	[23]
10	0.778	619.1270	C_26_H_28_O_16_	Quercetin glycoside 3	[M + Na]^+^	−0.07	[23]
11	0.445	628.1872	C_27_H_30_O_16_	Rutin	[M + NH_4_]^+^	0.02	[26]
12	8.202	493.1341	C_21_H_20_O_12_	Isoquercetin	[M + C_2_H_5_]^+^	−0.1	[26]
13	0.45	635.1970	C_27_H_30_O_15_	Kaempferol-3-O-rutinoside	[M + C_3_H_5_]^+^	0.07	[23]
14	0.728	313.1071	C_15_H_12_O_5_	Naringenin	[M + C_3_H_5_]^+^	−0.16	[23]

## Data Availability

Data is contained within the article.
